# Direct Incorporation of [^11^C]CO_2_ into Asymmetric [^11^C]Carbonates

**DOI:** 10.1155/2018/7641304

**Published:** 2018-12-10

**Authors:** Abdul Karim Haji Dheere, Salvatore Bongarzone, Dinah Shakir, Antony Gee

**Affiliations:** Department of Chemistry and Biology, School of Biomedical Engineering and Imaging Sciences, King’s College London, King’s Health Partners, St. Thomas’ Hospital, London, UK

## Abstract

A novel carbon-11 radiolabelling methodology for the synthesis of the dialkylcarbonate functional group has been developed. The method uses cyclotron-produced short-lived [^11^C]CO_2_ (half-life 20.4 min) directly from the cyclotron target in a one-pot synthesis. Alcohol in the presence of base trapped [^11^C]CO_2_ efficiently forming an [^11^C]alkylcarbonate intermediate that subsequently reacted with an alkylchloride producing the di-substituted [^11^C]carbonate (34% radiochemical yield, determined by radio-HPLC) in 5 minutes from the end of [^11^C]CO_2_ cyclotron delivery.

## 1 Introduction

Positron emission tomography (PET) is an imaging technique able to detect and monitor specific target proteins *in vivo* [1–5]. The use of PET imaging has advanced in the last few decades to become a valuable tool in clinical diagnostics, medical research, and drug discovery [6–8]. PET relies on the use of tracer amounts of imaging probes (radiotracers). The administration of radiotracers allows the biochemical process to be imaged and quantified *in vivo* without manifestation of pharmacological or toxicological effects [9–13].

Carbon-11 (^11^C) is one of the most common radionuclides used for the synthesis of PET radiotracers. The short half-life of ^11^C (20.4 min) makes it an attractive radionuclide as it enables the collection of a sufficient amount of PET data while keeping the subject radiation dose and exposure time to minimum. Furthermore, it allows orthologous substitution with carbon-12 in biologically active molecules with no alteration of the parent molecule’s physicochemical and pharmacological properties. Carbon-11 is commonly produced in the form of [^11^C]carbon dioxide ([^11^C]CO_2_) [14, 15]. [^11^C]CO_2_ is usually converted into more reactive secondary precursors such as [^11^C] methyl iodide ([^11^C]CH_3_I), [^11^C]carbon monoxide ([^11^C] CO), and [^11^C]phosgene ([^11^C]COCl_2_) [16–19]. As these multistep conversion processes are time-consuming, the use of [^11^C]CO_2_ for directly radiolabelling functional groups is highly attractive.

[^11^C]CO_2_ is a weak electrophile with an affinity for electron-donating reagents such as amines and organometallics [20]. However, due to the thermodynamic and kinetic properties of [^11^C]CO_2_, it has high activation energy which requires the use of highly reactive reagents, temperatures, pressures, or the presence of a catalyst [21–23]. Nevertheless, the primary synthon, [^11^C]CO_2_, has been deployed successfully for the synthesis of ^11^C-compounds that contain carbonyl groups such as [^11^C]carbamates [24, 25], amide [26], and [^11^C]ureas [23, 27–29]. However, the radiolabelling of the carbonyl group of carbonates from [^11^C]CO_2_ has not yet been established. To date, the synthesis of [^11^C]carbonates has relied on the use of [^11^C]COCl_2_ which is produced from a multistep process starting from cyclotron-produced [^11^C]CO_2_ or [^11^C]CH_4_, conversion to [^11^C]CCl_4_ and then to [^11^C]COCl_2_ [30, 31]. Although this ^11^C-carbonate reaction is rapid and efficient, routine production of [^11^C]COCl_2_ requires multistep syntheses and specialized equipment, thereby restricting its widespread use [30, 31].

As the carbonate functional group is found in prodrug compounds as well as being an intermediate in organic synthesis [32–35], we aimed at developing a simple and robust radiolabelling methodology that uses [^11^C]CO_2_ for the synthesis of [^11^C]carbonates. Here we present a rapid, one-pot radiosynthetic strategy using [^11^C]CO_2_ directly from the cyclotron, avoiding the need for specialized equipment and multistep syntheses.

## 2 Materials and Methods

All purchased chemicals were used without further purification. Chemicals were purchased in highest available purity from Sigma-Aldrich and Alfa Aesar and used as received (>99 % purity). All solvents were purchased as anhydrous in highest available purity (>99.8 % purity) from Sigma-Aldrich.

[^11^C]CO_2_ was produced by a Siemens RDS112 cyclotron (St Thomas’ Hospital, London, United Kingdom) via the ^14^N(p,*α*)^11^C nuclear reaction. Typical irradiation time for exploratory work was 1 minute, 10 *μ*A, bombardment typically yielding ca. 300 MBq [^11^C]CO_2_ at end of cyclotron bombardment. Radiolabelling reactions were performed in a 1.5 mL screw top vial with a “V” internal shape. HPLC analysis was performed on an Agilent 2060 Infinity HPLC system with a variable wavelength detector (254 nm was used as default wavelength) [10] An Agilent Eclipse XDB-C18 reverse-phase column (4.6 × 150 mm, 5 *μ*m) was used at a flow rate of 1 mL/min and H_2_O/MeOH (HPLC-grade solvents with 0.1 % TFA) gradient elution (flow rate: 1 mL/min, 0–2 min: 5 % MeOH, 2–11 min: 5 to 95% MeOH linear gradient, 11–13 min: 90 % MeOH, 13–14 min: 90% to 5% MeOH linear gradient, and 14–15 min: 5 % MeOH). The RCY was estimated by radio-HPLC and defined as the area under the ^11^C-product peak expressed as a percentage of the total ^11^C labelled peak areas observed in the chromatogram. Molar radioactivity was calculated from analytical HPLC sample of 25 *μ*L. A calibration curve of known mass quantity versus HPLC peak area (254 nm) was used to calculate the mass concentration of the 25 *μ*L radiolabelled compound. The identity of the radiolabelled compound peak was confirmed by HPLC coinjection of a nonradioactive reference compound and yielded a single peak.

## 3 Results and Discussion

**Figure 1:**
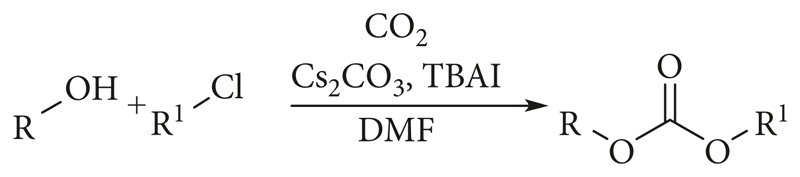
Method by Salvatore et al. [21–23] for the synthesis of carbonates using nonradioactive CO_2_.

As the starting point, we selected the method developed by Salvatore et al. [21–23] (Figure 1) for the synthesis of carbonates. The established method used nonradioactive CO_2_, an alcohol derivative, and benzylchloride (BzCl) in the presence of Cs_2_CO_3_, TBAI in DMF to produce the corresponding carbonate derivative efficiently. By substituting CO_2_ with [^11^C]CO_2_ and applying the same reaction conditions, the synthesis of di-substituted [^11^C]carbonates was investigated.

[^11^C]CO_2_ was trapped in isopropyl alcohol in the presence of Cs_2_CO_3_, forming an [^11^C]isopropylcarbonate intermediate that subsequently reacted with BzCl to produce [^11^C]benzyl isopropyl carbonate ([^11^C]**1**) in a moderate radiochemical yield (RCY). The RCY is the nonisolated radiochemical yield determined by radio-HPLC analysis of the crude product of 24% (Table 1, entry 1). Interestingly, almost all the cyclotron-produced [^11^C]CO_2_ was trapped within the reaction mixture at room temperature (>95%); any unreacted radioactive [^11^C]CO_2_ was immobilized on an ascarite trap connected to the vial vent needle. The trapping efficiency is the amount of radioactivity trapped in the reaction vial as a percentage of the overall radioactivity produced by the cyclotron.

In an attempt to increase the RCY, Cs_2_CO_3_ was replaced with Cs_2_SO_4_ (Table 1, entry 2). The trapping efficiency of [^11^C]CO_2_ dropped significantly from 95.2% to 1.5%. Since Cs_2_CO_3_ contributed towards the trapping of [^11^C]CO_2_ efficiently, we investigated whether the Cs^+^ or the CO_3_^2-^ ion was responsible for the high [^11^C]CO_2_-trapping efficiency. Of a number of caesium bases explored (Table 1, entries 3–5), CsI and CsF trapped only minute amounts of [^11^C]CO_2_ (4% and 34%, respectively), indicating that the basicity of the reaction mixture had a major effect on trapping efficiency. The results can be explained by the ability of a strong base to deprotonate the alcohol present in the reaction mixture enabling it to react with [^11^C]CO_2_ to form a ^11^C radiolabelled intermediate. The importance of CO_3_^2−^ was then explored by comparing Cs_2_CO_3_ with other carbonate bases (K_2_CO_3_ and CaCO_3_, Table 1, entries 6 and 7). The trapping efficiencies were extremely low for both reagents. High trapping in the reaction mixture with Cs_2_CO_3_ is therefore most likely due to its superior solubility in organic solvents.

In a further attempt to increase the RCY of [^11^C]**1**, a number of aprotic solvents were screened (CH_3_CN and DMSO, Table 1, entries 8 and 9). However, these solvents did not produce [^11^C]**1**, and the trapping efficiency was poor (20% and 65%, respectively). Reaction dependency on temperature was subsequently examined. The RCY of [^11^C]**1** improved from 24% to 33% by increasing the reaction temperature from 25°C to 65°C (Table 1, entry 10). Increasing the temperature to 100°C promoted the product formation and resulted in the highest observed RCY (82%, Table 1, entry 11). This might be rationalised by an increase in Cs_2_CO_3_ solubility at higher temperatures. However, due the presence of Cs_2_CO_3_ as a reagent, low molar activities (*A*_m_) were observed. The low *A*_m_ (2 GBq/*μ*mol in this case) is likely due to release of nonradioactive CO_2_ from Cs_2_CO_3_. CO_3_^2−^ deprotonates the alcohol to form HCO_3_^−^, which at high temperature has the potential to decompose releasing nonradioactive CO_2_ causing isotopic dilution and low *A*_m_ of the [^11^C]CO_2_. We therefore focused on improving *A*_m_ by substituting Cs_2_CO_3_ with an alternative base.

1,8-diazabicyclo[5.4.0]undecene (DBU) is a basic amine that has been shown to retain [^11^C]CO_2_ in organic solutions [26]. Replacing Cs_2_CO_3_ with DBU (Table 2, entry 1) resulted in [^11^C]**1** formation, but with low RCY (6%). The low RCY could be due to DBU being unable to deprotonate isopropyl alcohol efficiently. We opted for a stronger base, NaH, which was able to deprotonate the isopropyl alcohol. Using a ratio of 1 : 1 NaH : isopropanol (equiv.) at 100°C, [^11^C]**1** was obtained with an RCY of 26% (Table 2, entry 3). Decreasing the temperature from 100°C to 60°C slightly improved the RCY (31%, Table 2, entry 4). [^11^C]1 was produced with a molar activity (A_m_) of 10–20 GBq/*μ*mol. This is because short cyclotron bombardments (1 minute) and low beam currents (5–10 *μ*A) were used (0.3 GBq). In clinical productions at our facility, cyclotron bombardment times of 50 minutes and beam currents of 30 *μ*A are used to produce higher amounts of radioactivity (typically 60 GBq). It is therefore estimated that this would increase the Am to > 50 GBq/μmol at end of synthesis. Decreasing the ratio of NaH : isopropanol (from 1 : 1 to 0.5 : 1) reduced the RCY further to 18% (Table 2, entry 5). Increasing the ratio NaH : isopropanol 2 : 1 did not produce the target product (Table 2, entry 6). Increasing the amount of TBAI to 3 equiv. or removing it completely also did not improve the RCY (Table 2, entries 7 and 8).

## 4 Conclusions

In conclusion, we have developed a radiolabelling methodology for the synthesis of [^11^C]carbonates using [^11^C]CO_2_ directly from the cyclotron. The carbonate [^11^C]**1** was synthesized by bubbling [^11^C]CO_2_ into a solution containing alkylchloride, alcohol, and a base in DMF. The choice of the base was critical for maximising the RCY and *A*_m_. The first protocol uses Cs_2_CO_3_ and produces the target ^11^C radiolabelled product in a high RCY and low *A*_m_. The second strategy, which uses NaH, produced [^11^C]**1** in high *A*_m_ and moderate RCY. These methodologies are a simple and practical alternative to ^11^C-phosgene for the synthesis of ^11^C-carbonates. ^11^C-phosgene synthesis is technically challenging to implement and requires the use of specialist equipment. The developed strategies described here use readily available labware and converts [^11^C]CO_2_ directly to [^11^C]carbonates in rapid synthesis times.

## Figures and Tables

**Table 1:** Optimisation of [^11^C]**1** synthesis.

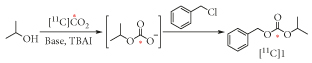					

Entry^a^	Base	Trapping efficiency (%)	Temperature (°C)	Solvent	RCY (%)^b^
1	Cs_2_CO_3_	95.2	25	DMF	24
2	Cs_2_SO_4_	1.5	25	DMF	0
3	CsI	4.3	25	DMF	5
4	CsF	33.5	25	DMF	0
6	K_2_CO_3_	10	25	DMF	0
7	CaCO_3_	0	25	DMF	0
8	Cs_2_CO_3_	20	25	CH_3_CN	0
9	Cs_2_CO_3_	65	25	DMSO	0
10	Cs_2_CO_3_	>95%	65	DMF	33
11^c^	Cs_2_CO_3_	>95%	100	DMF	82, 74

a Reaction conditions: isopropanol (22 *μ*mol), Cs_2_CO_3_ (66 *μ*mol), TBAI (66 *μ*mol), and organohalide (66 *μ*mol) in 500 *μ*L DMF, 10 mins from end of delivery (EOD) (*n* = 1).

b The nonisolated radiochemical yield determined by radio-HPLC analysis of the crude product.

c *n* = 2.

**Table 2:** Optimisation of [^11^C]**1** synthesis using alternative bases.

Entry^a^	Base (eq)	TBAI (eq)	Temp (°C)	RCY (%)y^b^
1^c^	DBU (3)	3	100	6
2^c^	DBU (3)	—	100	0
3	NaH (1)	1	100	26
4^d^	NaH (1)	1	60	31 ± 2
5^c^	NaH (0.5)	1	60	18
6	NaH (2)	1	60	0
7^c^	NaH (0.5)	—	60	6
8	NaH (1)	3	60	7

a Isopropanol (1 equiv., 22 *μ*mol), BzCl (3 equiv.), TBAI (1−3 equiv.), and base (1−3 equiv.) in 500 *μ*L DMF reaction time 5 mins from EOD.

b The nonisolated radiochemical yield determined by radio-HPLC analysis of the crude product.

c Reaction time of 10 mins from EOD.

d *n* = 3.
